# Considering Glucagon-like Peptide-1 Receptor Agonists (GLP-1RAs) for Weight Loss: Insights from a Pragmatic Mixed-Methods Study of Patient Beliefs and Barriers

**DOI:** 10.3390/healthcare14020186

**Published:** 2026-01-12

**Authors:** Regina DePietro, Isabella Bertarelli, Chloe M. Zink, Shannon M. Canfield, Jamie Smith, Jane A. McElroy

**Affiliations:** 1 Department of Family and Community Medicine, University of Missouri, Columbia, MO 65211, USA; maltagliaticm@health.missouri.edu (C.M.Z.); canfieldsm@health.missouri.edu (S.M.C.);; 2 School of Medicine, University of Missouri, Columbia, MO 65211, USA

**Keywords:** weight loss medication, weight management, obesity, patient centered care, patient perspectives, mixed methods research

## Abstract

**Highlights:**

**What are the main findings?**

- Patients expressed uncertainty about eligibility, long-term safety, and treatment expectations, underscoring the need for proactive clinician communication and shared decision-making.
- Cost and insurance coverage were the most significant barriers to initiating GLP-1RA therapy, with recent policy changes and FDA restrictions on compounding further limiting access.

**What are the implications of the main findings?**

- Clinicians should lead conversations about GLP-1RA therapy, clarify benefits and risks, and document prior weight loss efforts to support insurance approval.
- Addressing cost barriers and fostering patient confidence in treatment decisions are critical to reducing disparities and improving uptake of obesity pharmacotherapy.

**Abstract:**

**Background/Objective**: Glucagon-like peptide-1 receptor agonists (GLP-1RAs) have received widespread attention as effective obesity treatments. However, limited research has examined the perspectives of patients contemplating GLP-1RAs. This study explored perceptions, motivations, and barriers among individuals considering GLP-1RA therapy for obesity treatment, with the goal of informing patient-centered care and enhancing clinician engagement. **Methods**: Adults completed surveys and interviews between June and November 2025. In this pragmatic mixed-methods study, both survey and interview questions explored perceived benefits, barriers, and decision-making processes. Qualitative data, describing themes based on the Health Belief Model, were analyzed using Dedoose (version 9.0.107), and quantitative data were analyzed using SAS (version 9.4). Participant characteristics included marital status, income, educational attainment, employment status, insurance status, age, race/ethnicity, and sex. Anticipated length on GLP-1RA medication and selected self-reported health conditions (depression, anxiety, hypertension, heart disease, back pain, joint pain), reported physical activity level, and perceived weight loss competency were also recorded. **Results**: Among the 31 non-diabetic participants who were considering GLP-1RA medication for weight loss, cost emerged as the most significant barrier. Life course events, particularly (peri)menopause among women over 44, were commonly cited as contributors to weight gain. Participants expressed uncertainty about eligibility, long-term safety, and treatment expectations. Communication gaps were evident, as few participants initiated discussions and clinician outreach was rare, reflecting limited awareness and discomfort around the topic. **Conclusions**: Findings highlight that individuals considering GLP-1RA therapy face multifaceted emotional, financial, and informational barriers. Proactive, empathetic clinician engagement, through validation of prior efforts, clear communication of risks and benefits, and correction of misconceptions, can support informed decision-making and align treatment with patient goals.

## 1. Introduction

The surge in demand for and media coverage surrounding glucagon-like peptide-1 receptor agonist (GLP-1RA) medications, such as semaglutide (e.g., Wegovy, Ozempic) and tirzepatide (e.g., Zepbound, Mounjaro), has prompted a growing number of individuals with obesity to consider these therapies for weight loss [1,2,3]. Currently, approximately 42% of Americans meet the diagnostic criteria for obesity, and an estimated 12% have used a GLP-1RA for weight management, underscoring the widespread adoption of these medications as effective pharmacologic interventions [4,5,6,7]. As public awareness and accessibility increase, GLP-1RA use is expanding beyond individuals with chronic conditions to include those with limited prior experience using medications for long-term health management [1].

Numerous studies have explored the experiences of current or former GLP-1RA users [8,9,10,11,12,13], including motivations for initiation, prior weight loss strategies, risk perceptions, and anticipated benefits; however a gap remains in understanding the perspectives of individuals who are considering, but have not yet initiated, GLP-1RA therapy. Al-though not identical to our study population, a cross-sectional study in the United Kingdom compared views of adult non-users and users about their knowledge, attitudes, and perceptions of GLP-1RAs, finding that non-users were more skeptical about safety and more likely to believe that risks outweigh benefits [14]. Similarly, a study in Saudi Arabia reported limited awareness of GLP-1RA medication among residents of Hail, Riyadh, and Al-Ahsas. Refusals to consider these medications were primarily driven by fear of side effects, reluctance to take medication, and preference for diet and exercise to manage weight [15]. The Pew Research Center also analyzed how Americans view GLP-1RA medication and noted concerns about safety, long-term use, and perceptions of pharmacologic weight loss as a “shortcut” [16]. To our knowledge, no studies have explored the perspectives and experiences of U.S. adults considering GLP-1RA therapy for weight management.

Concurrent with patients’ interest in GLP-1RA medications, clinicians demonstrate heterogeneous levels of familiarity with obesity guidelines and divergent prescribing behaviors for GLP-1RA therapies. This is influenced by varying degrees of confidence, perceived benefit, degree of patient ownership in managing obesity and administrative barriers [8,17,18,19,20,21]. For example, cost and insurance coverage were noted as predominant barriers to prescribing, with Garvey’s study reporting 81% of surveyed physicians citing this barrier [18,19].

The Health Belief Model (HBM) offers a robust conceptual framework for examining patient perceptions, motivations, and barriers associated with GLP-1RA medication use for weight loss [22]. The model’s core constructs of perceived susceptibility, severity, benefits and barriers, cues to action, and self-efficacy are directly applicable to understanding why individuals may choose to initiate GLP-1RA therapy. For instance, perceived susceptibility and severity relate to how patients view their obesity-related health risks, while perceived benefits weigh the expected advantages of medication versus previous weight loss attempts. Perceived barriers, such as cost, side effects, and stigma influence willingness to try GLP-1RA medication. Self-efficacy captures confidence in managing the medication regimen and lifestyle changes. Previous applications of the HBM in obesity and medication adherence research underscore its predictive value in shaping behavioral intentions and actual health behaviors [23]. The purpose of this study is to apply the HBM to examine the perceptions of individuals considering GLP-1RA therapy for weight loss, identify themes in their decision-making process and compare these to the outcomes of current users, as reported in the literature. This study aimed to explore lived experiences, perceptions, and barriers among U.S. adults considering GLP-1RA therapy for weight management. Rather than testing a hypothesis, our goal was to understand patient perspectives to inform clinician–patient communication and improve access to therapy.

## 2. Materials and Methods

This study employed an explanatory convergent mixed methods design, guided by the Health Belief Model. After providing informed consent, participants were categorized into six distinct groups based on their experiences with GLP-1 receptor agonist (GLP-1RA) medications: those considering therapy (Group 1), those on therapy for ≤3 months (Group 2), those on therapy for >3 months without achieving their weight-loss goal (Group 3), those on therapy for >3 months with weight-loss goal success (Group 4), those who discontinued therapy after achieving their weight-loss goals (Group 5), and those who discontinued therapy without achieving their weight-loss goals (Group 6). This manuscript focuses on Group 1 participants, who were considering GLP-1RA therapy for weight loss. All participants first completed an online survey designed to assess their perceptions, beliefs, and experiences related to GLP-1RA treatment. Subsequently, they participated in a semi-structured interview to further explore and contextualize their responses, providing deeper insight into their decision-making processes and attitudes toward GLP-1RA therapy.

### 2.1. Recruitment Strategy

To be eligible for this study, participants had to meet the following criteria: age ≥ 18 years; no history of pancreatitis, type 2 diabetes, bariatric surgery, or personal/family history of MEN2 syndrome or medullary thyroid cancer; and willing to complete a semi-structured interview.

Participants were recruited using three distinct strategies. First, individuals prescribed GLP-1RA medication were identified from prescription refill requests from the Family and Community Medicine clinics. These potential participants were mailed an introductory letter describing the study, which included a URL linking to a REDCap survey containing eligibility screening and consent forms. The letter also informed recipients that they would be contacted by telephone to discuss participation. The second recruitment strategy utilized the Participant Registry for Research and Recruitment Core (PRRRCore), which distributed IRB-approved email communications (IRB# 2098619). These emails contained a brief description of the study and a link to the REDCap eligibility screening, contact information, and consent form. The third strategy involved ResearchMatch, a national health volunteer registry, through which individuals received an introductory message and a link to the REDCap forms [24]. All potential participants were contacted by telephone to answer questions and review the elements of informed consent. Once consent was obtained, participants were emailed a REDCap link to complete the study survey and a W-9 form for compensation. After survey completion, participants were contacted to schedule an interview. All enrolled participants provided informed consent in accordance with the principles set forth by the Declaration of Helsinki. The University of Missouri institutional review board (IRB #2125541) approved this study. Participants received a USD 20 e-gift card upon interview completion.

### 2.2. Quantitative Survey

Participants completed a 30-item survey. Survey data included age (in years) grouped into four categories (<25, 25–44, 45–64, >65), sex (male, female), comorbidities (such as cardiovascular disease, depression/anxiety, back/joint pain), race (White, Black, Hispanic, another race) and annual income (<USD 40K, USD 40K–59K, USD 60K–99K, USD 100K–149K, >USD 149K). Informed by HBM constructs and the current literature on GLP-1RA usage, the remaining tailored questions explored participants’ awareness and perceptions of GLP-1RAs, motivation for use, perceived concerns or barriers, prior weight loss experiences, cost and accessibility, personal health goals, physical activity assessment using International Physical Activity Questionnaire (IPAQ), Perceived Competency Scale (PCS) for weight loss (mean values: low < 4 points, moderate 4–5 points and high > 5 points) and long-term considerations of medication use. The survey was piloted before it was administered to the study participants. Descriptive analyses were conducted using SAS version 9.4 (SAS Institute Inc., Cary, NC, USA) [25].

### 2.3. Qualitative Interviews

The semi-structured interviews were organized according to the HBM constructs and prompted participants to share their experiences related to perceptions, motivations, and barriers associated with GLP-1RA medication use and weight loss (Table S1) [22]. Interviews were conducted by a team of four trained interviewers (JM, RD, SC, EM), all affiliated with the Department of Family and Community Medicine. Each interviewer had prior experience in qualitative interviewing. To avoid potential bias, any patient previously seen by RD was interviewed by a different team member. Given the limited peer-reviewed literature on this topic and the relatively homogeneous nature of this study, with a narrow focus on perceptions of GLP-1RA use, we aimed to conduct 10–20 interviews per group [26].

All interviews were audio recorded using Microsoft OneNote and/or Zoom. Transcripts were cleaned by JM, CM, and RD through a process of listening to the recordings and cross-checking the OneNote- and Zoom-generated transcripts. We employed conventional content analysis to explore responses to questions about the use of GLP-1RA medication for weight loss [27]. To ensure methodological transparency and analytic rigor, we adhered to the Consolidated Criteria for Reporting Qualitative Research (COREQ-32) [28]. Although the Health Belief Model (HBM) informed the development of the interview guide and provided a conceptual lens for interpreting findings, the coding process itself was inductive. This approach is well-suited to areas with limited prior research, allowing themes to emerge directly from participant narratives rather than being imposed by a pre-existing theoretical framework. Transcripts were imported into Dedoose (Version 9.0.107), a cloud-based platform for qualitative and mixed-methods analysis, to facilitate coding and codebook development. After transcribing 20 interviews, an initial codebook was developed by co-authors RD and JM, who bring complementary multidisciplinary perspectives to the analysis [29]. RD, with expertise in clinical practice, and JM, with a background in behavioral science and public health research, contributed distinct viewpoints that enriched the interpretation of participant responses and enhanced the depth and rigor of the coding framework. Each transcript was independently reviewed and coded by two members of the research team (BB, CM, JM), who were blinded to each other’s coding to enhance analytical rigor. Following independent coding, the coders met to reconcile discrepancies, and code definitions were revised to improve clarity and consistency, as needed, throughout the entire process. Dedoose automatically time-stamped each coded excerpt, enabling documentation of coding activity and internal audit procedures. To enhance the rigor, intercoder reliability was assessed using Dedoose’s Testing Center feature. The pooled kappa summarizes rater agreement across multiple codes to evaluate agreement beyond chance.

## 3. Results

**Figure 1:**
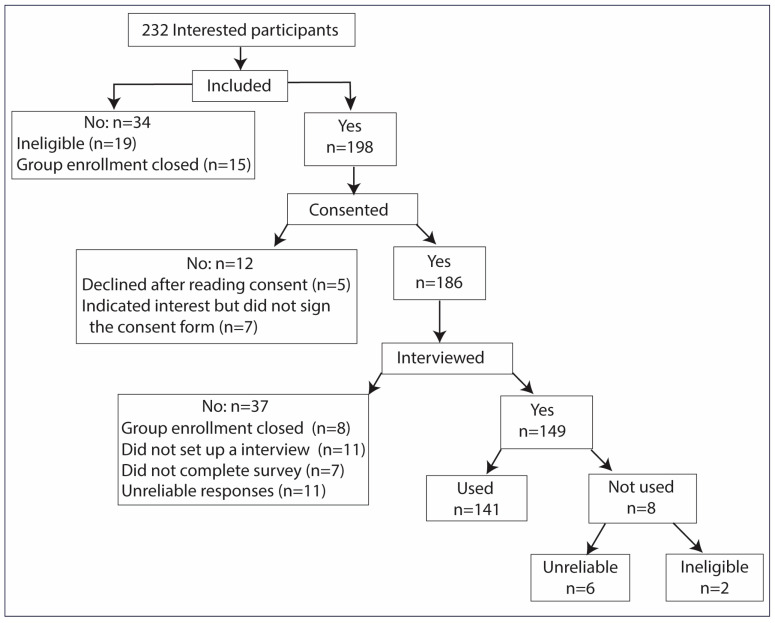
Participant flow diagram.

Between June and November 2025, among the 232 participants who expressed interest in the study, 80% (*n* = 186) provided informed consent and were enrolled. Of these, 37 consented but were not interviewed; 18 did complete the survey or schedule an interview, and another 8 were excluded because enrollment closed for that group. A subset of 11 participants recruited via ResearchMatch who provided consent and completed the survey exhibited patterns suggesting automated or unreliable responses (e.g., multiple surveys completed within minutes, implausible data such as a weight of 20 lbs, or highly improbable medication use). Eight interviews were excluded from analysis: six due to implausible responses (unreliable) and two due to ineligibility identified during the interview. The final analytic sample included 141 interviews. See Figure 1 for details of the enrollment process.

To assess the reliability of the qualitative coding, a subset of 10 codes from a random sample of 59 transcripts were independently coded by JM (lead) and CM. Cohen’s Kappa was calculated for each code, and the pooled Kappa score was 0.81, indicating excellent agreement (scores above 0.75 are considered excellent) [30,31,32].

For this analysis, 31 participants (Group 1: Thinking about using GLP-1RA as weight loss strategy) were interviewed (length: 12–49 min, mean 24 min). The mean age of this group was 42 years (19–79 years), and the majority were White and multiracial (74%, 13%), female (71%), in the labor force (85%), had private insurance (80%), and married (64%). Almost one-third (37%) of the participants earned less than USD 60,000 (Table 1).

The following paragraphs describe six major study themes: (1) a personal history of weight-related challenges, (2) motivation to prevent or address obesity, (3) understanding the long-term consequences of obesity (4) perceived benefits of GLP-1RA therapy, (5) perceived benefits of GLP-1RA use for co-morbidities, and (6) barriers to using GLP-1RA.

### 3.1. Personal Histories of Weight-Related Challenges

The participants’ narratives often included personal histories of weight gain, shaped by factors such as lifelong struggles with obesity, sedentary work environments, having children, aging, and menopause. These contextual experiences informed how these individuals perceived their susceptibility to obesity-related conditions, the severity of their current health status, the potential benefits and barriers of GLP-1RA therapy and their interactions with care professionals. For example, one 39-year-old White female described the confusion around her inability to lose weight despite professional support: “*When I’m with a dietitian, she’s like, I don’t know why you’re not losing weight other than you might be going into menopause*.” Another participant, a 47-year-old White male, reflected on how life transitions contributed to gradual weight gain over nearly two decades: “*Then once I got married, then the weight really started going on. I was in graduate school, and wasn’t active, and then we had kids, and I was even less active, life stress, and so…the weight gain has been probably pretty gradual for 20 years now. It’s been pretty steady.*” These personal accounts highlight how weight gain is often experienced as a cumulative and multifactorial process deeply intertwined with life events and physiological changes.

### 3.2. Motivation to Prevent or Address Obesity

Belief in the potential to prevent obesity-related health problems emerged as an important motivator among participants considering GLP-1RA medication. A 34-year-old White female shared, “*My entire family has diabetes, and I haven’t been diagnosed with it yet. I’m trying to stop from being diagnosed diabetic,*” while another 59-year-old White female noted, “*I’m not big on medicine and surgeries… but I also don’t want to be on cholesterol medicine and high blood pressure medicine… I see it [GLP medication] as it could be a preventative.*”

### 3.3. Understanding the Long-Term Consequences of Obesity

The perceived seriousness of obesity and its long-term consequences also emerged as a key theme among those considering GLP-1RA medication. Concerns about future health risks spanned age groups. A 21-year-old White female, for example, expressed fear about losing mobility due to her sedentary lifestyle: “*I do have a sedentary lifestyle. I don’t really have a whole lot of control over it. And it’s very scary to be afraid of losing the mobility I do have*.” Similarly, a 46-year-old Hispanic female emphasized the potential for chronic illness and premature death as a decisive factor in considering weight loss interventions: “*My risk of earlier death or more chronic illness or something, if that is to the point of concern, then I think that is the number one thing that would really tip me in that decision to start to be able to lose weight*.” These reflections illustrate how some participants’ recognition of the severity of obesity, both in terms of physical limitations and life-threatening outcomes, strongly influenced their openness to considering treatment options such as GLP-1RA therapy.

### 3.4. Perceived Benefits of GLP-1RA Therapy

The participants identified numerous perceived benefits of GLP-1RA therapy, particularly in association with the presumed weight loss. They connected this outcome to reduced health risks, improved overall well-being, and enhanced quality of life. A 22-year-old White female described her motivation as being rooted in her family history: “*I would say it’s always been on my mind because of my hereditary [risk] and being predisposed to cardiovascular disease. But now I feel I should try to lose weight to prevent that*.” Similarly, a 34-year-old White male emphasized the importance of maintaining health for family responsibilities: “*I’m just trying to get myself in better shape for my family. I’ve got a 3-year-old son. I’d like to be around for him*.” And a 46-year-old White female highlighted the broader physical and emotional benefits of weight loss: “*Obesity causes a lot of things in your body. It’s hard on your joints and on your heart. If you’re getting relief in other areas as well, that’s huge—because if it helps with your inflammation and your body hurts all the time, that’s why you don’t exercise. Hopefully, getting that weight off and helping with the inflammation means I’ll feel up to being more active. Being more active burns more calories, and that, along with getting your endorphins going, heightens your mood*.”

### 3.5. Perceived Benefits of GLP1-RA Use for Co-Morbidities

Among respondents who reported specific health conditions, (i.e., hypertension or heart disease (35%) and anxiety and/or depression (45%)), only one participant explicitly expressed an expectation that their condition would improve with GLP-1RA use. In contrast, two of the six participants with joint or back pain conveyed hope that weight loss would alleviate their discomfort. As one 79-year-old White male noted, “*I would like to get around a lot better. I’d like to be able to walk through a supermarket without using one of those electric scooters*.”

This response may reflect a broader public understanding that excess body weight is directly associated with musculoskeletal conditions, including osteoarthritis and back pain, whereas the connections between obesity and cardiovascular or mental health outcomes, though well-established in the scientific literature, are less widely recognized among the general population [33,34,35].

### 3.6. Barriers to Using a GLP1-RA

Numerous barriers were cited by Group 1 participants, likely contributing to their hesitancy in initiating GLP-1RA therapy, despite a shared recognition that weight loss was an important health priority. These barriers included eligibility, safety concerns, cost, and social perceptions that commonly deter individuals from initiating pharmacologic weight-management interventions [14,20,36,37,38].

#### 3.6.1. Eligibility 

Several participants questioned whether they were appropriate candidates for GLP-1RA use, expressing uncertainty about clinical eligibility or the appropriateness of treatment for individuals without diabetes. As one 30-year old Black female explained, “*I just think for its intended use, I don’t know that I fall into that category. I haven’t had any insulin resistance. So, I don’t know that it would be the smartest thing for me to do since I’m not in the target*.” This uncertainty aligns with studies showing that a limited understanding of indication criteria and a lack of provider guidance contribute to hesitancy and underuse of anti-obesity medications among individuals with overweight or obesity [8,14].

#### 3.6.2. Safety

Concerns about the medication’s safety and long-term effects were also prominent. A 51-year-old White female stated, “*I was hesitant early on because I felt like there wasn’t enough data about how this was affecting people long term*. Thisreflected broader public apprehension toward newly approved weight-loss drugs. Similarly, potential drug–drug interactions were cited as a deterrent, particularly among those taking chronic medications [36,39]. A 22-year-old White female shared, “*I’ll be honest, I’m on birth control and if there was a negative reaction to that, I would say my current medications are more important than weight loss.*”

#### 3.6.3. Financial

Financial barriers were salient across interviews, with cost and a lack of insurance coverage described as critical determinants of access [40,41]. Even in cases where employer-sponsored insurance was present, cost was cited as a concern by all but one participant, with financial constraints particularly pronounced among respondents earning less than USD 60,000 annually (36% of this group). No statistical differences by sex or race were noted. A 29-year-old Black female stated, “*I’m living paycheck to paycheck right now. So, if insurance can’t cover most of it, then I’m just gonna not do it because I can’t afford it*,” while a 41-year-old White female added, “*As long as it’s covered by insurance. I don’t mind if there’s like a copay or anything. I’m not paying for it out of pocket though.*” These concerns reflect well-documented disparities in access to GLP-1RAs driven by high out-of-pocket costs and inconsistent insurance reimbursement, particularly among lower-income and racially minoritized populations [40,41,42].

#### 3.6.4. Stigma

Finally, several participants described social and identity-related barriers, such as concerns about stigma or perceived inauthenticity in achieving weight loss through medical means. A 54-year-old White male remarked, “*I’d like to think that most people wouldn’t think I was cheating. Instead of going to the gym, I’m taking a medical evolution*,” while a 19-year-old White female expressed concern about how medication-related changes might affect her self-perception: “*I would worry that any changes in my appearance from the medication might not be recognized. So not having a solid picture of what I actually look like [makes me hesitant]*.” Such narratives echo findings that weight-loss stigma and moralized views of obesity treatment can lead individuals to perceive pharmacotherapy as “taking the easy way out,” further discouraging uptake [43,44,45].

### 3.7. Knowledge

Participants in this group demonstrated limited knowledge and mixed opinions regarding the safety, potential effects, and anticipated duration of GLP-1RA use, which can play a role in the decision-making process. When surveyed about perceived safety, males were more unconcerned than females (18% vs. 86%) although nearly one-quarter (23%) reported never having considered the issue. Most participants (approximately 75%) were unable to identify any potential side effects, and nearly one-third (32%) were unsure as to whether GLP-1RAs would be prescribed as a short-term aid or a lifelong therapy. This uncertainty aligns with recent evidence showing that public understanding of GLP-1RAs remains limited, particularly regarding their mechanism, long-term effects and duration of treatment, despite their increasing visibility in popular media [46,47].

While baseline knowledge was limited, social exposure and peer influence often acted as informal cues prompting reconsideration of GLP-1RA therapy. Participants frequently mentioned hearing about the medication through family, coworkers, or social media. A 56-year-old mixed-race female shared that she had “*seen some pretty incredible results for some of [her] family members*,” which led her to wonder if “*maybe this is the thing that will get me over that hump*” of constantly thinking about food and dieting. Similarly, a 47-year-old White female admitted, “*I am not immune from the social media influence that seems to point to dramatic changes for people who take it*,” while a 34-year-old White female described coworkers enthusiastically urging her to “*get on it as soon as you can*.”

These narratives illustrate how vicarious experiences and media exposure can shape perceptions of efficacy and safety, often filling an informational void left by limited professional guidance. Such findings parallel recent analyses showing that online communities and social media platforms have become dominant sources of information and misinformation about GLP-1RAs, amplifying enthusiasm while downplaying risks [48,49]. For some, these social comparisons intersect with age-related concerns about metabolism and weight maintenance. As one 54-year-old White male observed, “*metabolism and age has really kind of caught up with me within the course of the past five to maybe seven years*,” suggesting how personal experiences of aging may compound openness to pharmacologic solutions.

Collectively, these findings highlight a disconnect between widespread social awareness of GLP-1RAs and the relatively low level of clinical understanding among potential users. This imbalance underscores the need for proactive, evidence-based education to help individuals differentiate between anecdotal success stories and the medical realities of long-term pharmacologic weight management [14,19,48].

### 3.8. Weight Loss Confidence and Self-Efficacy

Self-efficacy, the confidence in one’s ability to initiate and sustain behaviors required for weight loss, influenced participants’ consideration of GLP-1RA medication within the framework of the Health Belief Model. On the standardized perceived competence scale, which closely reflects self-efficacy, this group scored in the lowest tertile (mean = 3.9), indicating limited confidence in their ability to achieve meaningful weight loss through personal effort alone compared to the other groups that had initiated GLP-1RA therapy [50,51]. This finding aligns with prior research showing that individuals with obesity frequently report diminished self-efficacy for weight control, particularly after repeated unsuccessful attempts at dieting or exercise [52,53].

Consistent with this interpretation, 90% of participants who were thinking about GLP-1RA medication reported having tried multiple weight loss strategies such as diet modification and exercise, and approximately one-third had previously used weight-loss medications without achieving sustained results. This pattern of repeated but unsuccessful effort likely reinforces perceptions of limited personal control and reduces motivation to engage in future attempts, a dynamic well-documented in behavioral models of obesity treatment [54,55]. Interestingly, even among those with objectively higher activity levels, as measured by the physical activity questionnaire (IPAQ), self-efficacy remained low. Although 32% of participants scored high on the IPAQ, suggesting they were already engaging in above-average physical activity, many still reported difficulty losing weight. However, self-efficacy varied among participants. One 56-year-old multiracial female demonstrated notable decisional confidence, stating, “*If I decide to do this, I would have a pretty open dialogue with a number of people who really matter to me. I have no one in my life who would discourage any path I choose to take*.” Her statement suggests a high level of confidence in managing her own health decisions, supported by positive social reinforcement, a factor that, according to the HBM, enhances self-efficacy through social persuasion and interpersonal cues to action [51,56].

## 4. Discussion

Our study captures patient viewpoints during a unique point in time when there are many people eligible to start GLP-1RA medications for obesity who have not yet tried them. As of December 2025, the World Health Organization (WHO) has recommended the use of GLP-1RAs for the treatment of obesity [57]. This paper can help elucidate why some patients are hesitant about starting this medication despite the medication’s proven success in treating obesity. Since our interviews were conducted in 2025, five years after semaglutide (NovoNordisk A/S, Plainsboro, NJ, USA) and two years after tirzepatide (Eli Lilly and Company, Indianapolis, IN USA) received FDA approval for the treatment of obesity, the data reflect a moment at which longitudinal safety data remained limited [4]. This context may have influenced participants’ concerns and hesitations regarding GLP-1RA therapy. Participants in our study expressed concerns about long-term health effects and insurance coverage, themes also reported among current GLP-1RA users [9,11]. However, our interviews revealed additional complexities, including questions about the value of GLP-1RAs in the context of societal stigma and limited clinician engagement. Despite these medications’ popularity and perceived effectiveness, participants noted fewer proactive discussions with healthcare providers than might be anticipated, given their widespread use.

### 4.1. Barriers to GLP-1RA Use

Cost emerged as a prominent theme across interviews and was consistently identified as a significant barrier to initiating GLP-1RA treatment. Participants expressed concern about affordability, particularly in light of recent decisions by private insurers to discontinue coverage for weight loss for non-diabetic patients starting in 2026. These policy changes, often justified by insurers as necessary to manage unsustainable costs, have shifted financial responsibility to employers or imposed behavioral prerequisites and preauthorization requirements [58,59]. For many individuals, especially those with limited financial resources, such changes represent a substantial obstacle to accessing treatment.

In response to these coverage gaps, some participants reported considering compounded versions of GLP-1RA medications, which are typically less expensive but may lack the rigorous regulatory oversight of FDA-approved products [60]. While compounding has historically offered an alternative for uninsured or underinsured patients, recent FDA actions to restrict compounding due to safety concerns have introduced new challenges. Patients who previously relied on compounded medications now face uncertainty regarding both affordability and availability, potentially exacerbating disparities in access to obesity treatment [40].

### 4.2. Opportunities for Clinician Engagement

Beyond cost and access, participants expressed uncertainty about their candidacy for GLP-1RA therapy, potential side effects, long-term safety, and treatment duration. These concerns highlight the need for proactive, clinician-initiated conversations that clearly outline the benefits, risks, and expectations regarding therapy. Our findings suggest opportunities for clinicians to support shared decision-making by acknowledging patients’ current health behaviors, such as exercise and dietary changes, before offering generalized lifestyle recommendations. Documenting prior weight loss efforts (e.g., diet, physical activity, previous medication use) in the medical record may also facilitate insurance approval for GLP-1RA therapy, which often requires evidence of unsuccessful conventional approaches [61].

Importantly, clinicians should not interpret a patient’s silence or lack of inquiry about GLP-1RA therapy as disinterest. As described by the participants, such silence may reflect uncertainty about eligibility, limited awareness of therapeutic benefits, or discomfort initiating the conversation. Given this ambiguity, clinicians should take the lead in introducing GLP-1RA as a potential treatment option, particularly when patients present with obesity-related health concerns or express interest in weight management. Prior research suggests that patients are more likely to pursue treatment when it is endorsed by their physician, underscoring the importance of clinician guidance in facilitating informed decisions [9,62]. Although GLP-1RAs offer several non-weight-related health benefits, participants’ awareness of these advantages was limited [63]. Clear communication about risks, expected outcomes, and potential adverse effects is essential to support patient confidence and informed decision-making [1].

### 4.3. Implications for Patient-Centered Care

Participants’ reflections on GLP-1RA therapy as a strategy to prevent obesity-related health conditions align with a robust body of literature demonstrating the link between obesity and chronic diseases, such as cardiovascular disease, type 2 diabetes, infertility, and increased mortality [64]. Notably, during the COVID-19 pandemic, obesity was identified as a major risk factor for severe outcomes, including hospitalization, ICU admission, and death [65,66]. A large-scale study involving over two million individuals found that GLP-1RA use, compared to usual care, was associated with a reduced risk of developing diabetes and other chronic conditions, highlighting its potential as a preventive therapy [67]. These findings illustrate how personal and familial health histories can shape motivation to pursue pharmacologic interventions aimed at disease prevention.

Participants’ perceptions of the benefits of GLP-1RA therapy are consistent with existing studies indicating that perceived health benefits. For example, preventing chronic disease or improving physical functioning can serve as strong motivators for weight loss and sustained health behavior change. Overall, participants’ views on the benefits of weight loss aligned with public health evidence showing that modifiable risk factors, including poor diet, physical inactivity, and obesity, contribute to nearly 50% of premature deaths in the United States [68,69,70]. Moreover, preventive health behaviors like weight management not only improve individual health outcomes but also reduce long-term healthcare costs compared to treating chronic conditions after onset [71,72].

This study also highlights the complex interplay between behavioral effort, perceived competence, and outcome expectations, emphasizing that self-efficacy is shaped not only by actions but also by the perceived success of those actions [52,54]. Low self-efficacy has been shown to predict reduced adherence to lifestyle interventions and hesitation to initiate new treatments, such as pharmacotherapy [54,55]. For some participants, GLP-1RA therapy represented an external solution compensating for perceived limitations in willpower or behavioral control, an expression of the Health Belief Model’s construct of low self-efficacy, or limited belief in one’s ability to take effective action toward change [51]. In this context, low perceived competence may reduce the likelihood of initiating treatment, even when perceived benefits are high.

Interestingly, self-efficacy and decisional confidence diverged. While many participants reported limited confidence in their ability to lose weight independently, some expressed high confidence in their autonomy to make treatment decisions. This form of decisional self-efficacy may enhance openness to pharmacologic interventions when they align with personal values and trusted social networks. Consistent with prior research, these findings underscore the need for interventions that not only provide accurate information about GLP-1RA therapy but also foster patients’ confidence in their ability to make and sustain informed health choices [68,73].

### 4.4. Future Work

This mixed-methods study provides a foundation for future research aimed at improving patient-centered GLP-1RA care. Future studies should intentionally include diverse populations, such as men, rural populations, and individuals from different socioeconomic and cultural backgrounds, to examine how perspectives vary across social and economic contexts. Additional research is needed to generate robust data on the long-term effects of GLP-1RA therapy and to develop strategies for effectively disseminating emerging information to patients seeking safety guidance. Finally, clinician-focused interventions should aim to identify best practices for initiating conversations about GLP-1RA medications with patients who have obesity, including those with and without co-morbidities; establish evidence-based guidelines for an optimal follow-up schedule; and standardize educational materials on nutrition and physical activity to support informed, patient-centered decision-making.

### 4.5. Limitations

The results of this study should be considered in light of its limitations. We wanted to capture a point in time when GLP-1RA medications were surging in use. Although this study was not limited to a single site, we relied on prescription refill information and clinician referrals within one health care system as the primary recruitment strategy. This approach may have introduced selection bias and limited the diversity of perspectives. Most participants were White and female, which reflects broader prescribing trends, as women are more likely to use a GLP-1RA medication for obesity alone [74].

## 5. Conclusions

These insights are essential for understanding patients’ perceptions of obesity and their openness to pharmacological interventions, such as GLP-1RA therapy. Findings from this study can inform clinical practice by helping providers address common questions, misconceptions, and concerns related to GLP-1RA use. Furthermore, the results support the development of tailored counseling strategies that proactively address potential barriers, enhance patient education, and promote informed decision-making. By acknowledging and addressing stigma associated with obesity and its treatment, these insights may also contribute to normalizing the use of pharmacotherapy as a legitimate and acceptable option for weight management.

## Figures and Tables

**Table 1:** Characteristics of participants thinking about using a GLP-1RA.

Characteristics	*N* = 31	Percent
Perception of Length		
Lifelong	4	13%
Short term	12	39%
Follow doctor’s advice	5	16%
Don’t know/Not sure	10	32%
Marital status *		
Never married	8	26%
Married/long-term relationship	20	64%
Widowed/divorced/separated	2	6%
Income *		
<USD 20,000	3	9%
USD 20,000 to 39,999	7	22%
USD 40,000 to 59,999	2	6%
USD 60,000 to 99,999	7	22%
USD 100,000 to USD 149,999	3	13%
USD 150,000 to 174,999	1	3%
USD 175,000 or higher	6	19%
Educational attainment		
High school or GED	0	
Some college or technical school—no degree	6	19%
2-year college degree/technical school degree	2	7%
4-year college degree	14	45%
Post-graduate work or degree	9	29%
Employment status		
Working full or part-time	26	85%
Retired	1	3%
Disabled or unable to work	1	3%
In school full time and not working	3	9%
Insurance status *		
Medicaid	2	7%
Medicare	1	2%
Multiple	2	7%
Private	24	80%
Self-pay	1	2%
Age (in years)		
18–24	4	13%
25–44	12	39%
45–64	12	39%
65+	3	9%
Race		
White	23	74%
Hispanic	1	3%
Multiracial	4	13%
Black	2	6%
Another race	1	3%
Sex		
Female	22	71%
Male	9	29%
Health conditions		
Depression/anxiety	10	32%
Depression only	0	0%
Anxiety only	4	13%
Neither depression nor anxiety	21	55%
High blood pressure (HBP) only	8	26%
Heart disease only	1	3%
HBP/heart disease	2	6%
Back pain only	2	6%
Joint pain only	1	3%
Back/joint pain	3	10%

* Insurance and marital status data were missing for 1 participant and income data were missing data for 2 participants.

## Data Availability

The raw data supporting the conclusions of this article will be made available by the authors on request.

## Supplementary Materials

The following supporting information can be downloaded at https://www.mdpi.com/article/10.3390/healthcare14020186/s1, Table S1: Participants considering GLP-1RA interview questions.

## Abbreviations

GLP-1RA	Glucagon-like peptide-1 Receptor Agonists
HBM	Health Belief Model
MEN2	Multiple Endocrine Neoplasia, type 2
REDCap	Research Electronic Data Capture
URL	Uniform Resource Locator
PRRRCore	Participant Registry for Research and Recruitment Core
IRB	Institutional Review Board
IPAQ	International Physical Activity Questionnaire
SAS	Statistical Analysis System
COREQ-32	Consolidated Criteria for Reporting Qualitative Research
FDA	Food and Drug Administration
COVID-19	Coronavirus Disease 2019
ICU	Intensive Care Unit
WHO	World Health Organization

## References

[1] Naveed M., Perez C., Ahmad E., Russell L., Lees Z., Maybury C. (2025). GLP-1 medication and weight loss: Barriers and motivators among 1659 participants managed in a virtual setting. Diabetes Obes. Metab..

[2] Heitmann B.L. (2025). The Impact of Novel Medications for Obesity on Weight Stigma and Societal Attitudes: A Narrative Review. Curr. Obes. Rep..

[3] Podolsky M.I., Raquib R., Shafer P.R., Hempstead K., Ellis R.P., Stokes A.C. (2025). Factors Associated with Semaglutide Initiation Among Adults with Obesity. JAMA Netw. Open.

[4] Aronne L.J., Horn D.B., le Roux C.W., Ho W., Falcon B.L., Gomez Valderas E., Das S., Lee C.J., Glass L.C., Senyucel C. (2025). Tirzepatide as Compared with Semaglutide for the Treatment of Obesity. N. Engl. J. Med..

[5] Bozick R., Donofry S.D., Rancano K.M. New Weight Loss Drugs GLP-1 Agonist Use and Side Effects in the United States. https://www.rand.org/pubs/research_reports/RRA4153-1.html.

[6] Montero A., Sparks G., Presiado M., Hamel L. KFF Health Tracking Poll May 2024: The Public’s Use and Views of GLP-1 Drugs. https://www.kff.org/health-costs/poll-finding/kff-health-tracking-poll-may-2024-the-publics-use-and-views-of-glp-1-drugs/.

[7] Centers for Disease Control and Prevention (CDC) Adult Obesity Facts. https://www.cdc.gov/obesity/adult-obesity-facts/index.html.

[8] Kaplan L.M., Gudzune K., Ard J., Kumar R., Ahmad N.N., Kan H., Sims T.J., Poon J.L., King-Concialdi K., Beusterien K. (2025). Perceptions of anti-obesity medications among people with obesity and healthcare providers in the US: Findings from the OBSERVE Study. Obesity.

[9] Ibsen C.K., Brostrom Kousgaard M., Olsen S., Christiansen A.L., Sandholdt C.T., Rorth R., Overbeck G. (2025). Patients’ experiences with GLP1-Ras—A systematic review. Scand. J. Prim. Health Care.

[10] Waldrop S.W., Johnson V.R., Stanford F.C. (2024). Inequalities in the provision of GLP-1 receptor agonists for the treatment of obesity. Nat. Med..

[11] Febrey S., Nunns M., Buckland J., Abbott R., Bethel A., Whear R., Boddy K., Melendez-Torres G.J., Coon J.T., Shaw L. (2025). What Are the Experiences, Views and Perceptions of Patients, Carers and Clinicians of Glucagon-like Peptide-1 Receptor Agonists (GLP-1 RAs)? A Scoping Review. Health Expect..

[12] McVay M.A., Moore W.S., Wilkins F.L., Jackson J.R., Robinson M.D. (2024). Patient perspectives on incretin-based weight loss medications and relationship with demographic factors. Obes. Sci. Pract..

[13] Plenn E., Amin D., Henry J., Leavitt G., Walker J., Soleymani T. (2025). A Qualitative Analysis of Patient Experiences Using Semaglutide 2.4 mg for Weight Loss. Obes. Sci. Pract..

[14] Auerbach N., Liu V.N., Huang D.R., Clift A.K., Al-Ammouri M., El-Osta A. (2025). What are community perspectives and experiences around GLP-1 receptor agonist medications for weight loss? A cross-sectional survey study in the UK. BMJ Public Health.

[15] Almughais E.S., Alshehri M.H., Alsatti M., Almatar A., Albladi F.H., Almomatin H.H., Alshammari N.M., Alshammari R. (2023). Awareness and Perception of Anti-obesity Medications Among Al-Ahsaa, Riyadh, and Hail in Saudi Arabia Populations. Cureus.

[16] Tyson A., Kikuchi E. How Americans View Weight-Loss Drugs and Their Potential Impact on Obesity in the U.S. https://www.pewresearch.org/science/2024/02/26/how-americans-view-weight-loss-drugs-and-their-potential-impact-on-obesity-in-the-u-s/.

[17] Smith M., Gallagher C., Weber D., Dietz W.H. (2023). Health care providers’ attitudes and counseling behaviors related to obesity. Obes. Sci. Pract..

[18] Yaseen A., Lahiri S.W. (2023). Health Care Provider Prescribing Habits and Barriers to Use of New Type 2 Diabetes Medications: A Single-System Survey Study. Clin. Diabetes.

[19] Garvey W.T., Mahle C.D., Bell T., Kushner R.F. (2024). Healthcare professionals’ perceptions and management of obesity & knowledge of glucagon, GLP-1, GIP receptor agonists, and dual agonists. Obes. Sci. Pract..

[20] Holtrop J.S., Tietbohl C., Perreault L., Connelly L., Smith P.C., Williams J. (2025). Primary care patient and practice member perspectives on weight loss medications: Challenges and opportunities. Front. Med..

[21] Trocchio L.L., Peters F. (2026). Taking back control: The experience of adults using semaglutide and tirzepatide for obesity treatment—A qualitative study. Obes. Pillars.

[22] Rosenstock I.M. (1966). Why people use health services. Milbank Mem. Fund. Q..

[23] Alyafei A., Easton-Carr R. (2024). The Health Belief Model of Behavior Change.

[24] Harris P.A., Scott K.W., Lebo L., Hassan N., Lightner C., Pulley J. (2012). ResearchMatch: A national registry to recruit volunteers for clinical research. Acad. Med..

[25] SAS Institute Inc. (2023). SAS® software, Version 9.4.

[26] Hennink M., Kaiser B.N. (2022). Sample sizes for saturation in qualitative research: A systematic review of empirical tests. Soc. Sci. Med..

[27] Hsieh H.F., Shannon S.E. (2005). Three approaches to qualitative content analysis. Qual. Health Res..

[28] Tong A., Sainsbury P., Craig J. (2007). Consolidated criteria for reporting qualitative research (COREQ): A 32-item checklist for interviews and focus groups. Int. J. Qual. Health Care.

[29] Kallio H., Pietilä A.M., Johnson M., Kangasniemi M. (2016). Systematic methodological review: Developing a framework for a qualitative semi-structured interview guide. J. Adv. Nurs..

[30] Landis J.R., Koch G.G. (1977). The measurement of observer agreement for categorical data. Biometrics.

[31] Cicchetti D.V. (1994). Guidelines, criteria, and rules of thumb for evaluating normed and standardized assessment instruments in psychology. Psychol. Assess..

[32] Fleiss J.L. (1971). Measuring nominal scale agreement among many raters. Psychol. Bull..

[33] Walsh T.P., Arnold J.B., Evans A.M., Yaxley A., Damarell R.A., Shanahan E.M. (2018). The association between body fat and musculoskeletal pain: A systematic review and meta-analysis. BMC Musculoskelet. Disord..

[34] Luppino F.S., de Wit L.M., Bouvy P.F., Stijnen T., Cuijpers P., Penninx B.W., Zitman F.G. (2010). Overweight, obesity, and depression: A systematic review and meta-analysis of longitudinal studies. Arch. Gen. Psychiatry.

[35] Schreurs L., Gies I., Van der Schueren B., De Cock D. (2025). Systematic literature review on the awareness of obesity in adults and children living with obesity, the general public and healthcare professionals. Int. J. Obes..

[36] Srivastava G., Apovian C.M. (2018). Current pharmacotherapy for obesity. Nat. Rev. Endocrinol..

[37] Wadden T.A., Chao A.M., Moore M., Tronieri J.S., Gilden A., Amaro A., Leonard S., Jakicic J.M. (2023). The Role of Lifestyle Modification with Second-Generation Anti-obesity Medications: Comparisons, Questions, and Clinical Opportunities. Curr. Obes. Rep..

[38] Saran A., Raisinghani R., Paliwal S., Sharma S. (2025). GLP-1R agonists: Recent advances, current gaps, and future challenges. Mol. Divers..

[39] Min J.S., Jo S.J., Lee S., Kim D.Y., Kim D.H., Lee C.B., Bae S.K. (2025). A Comprehensive Review on the Pharmacokinetics and Drug-Drug Interactions of Approved GLP-1 Receptor Agonists and a Dual GLP-1/GIP Receptor Agonist. Drug Des. Dev. Ther..

[40] Pearson S.D., Whaley C.M., Emond S.K. (2025). Affordable access to GLP-1 obesity medications: Strategies to guide market action and policy solutions in the US. J. Comp. Eff. Res..

[41] Herges J.R., Neumiller J.J., McCoy R.G. (2025). Navigating Cost and Access Barriers for Medications in the Treatment of Obesity: A Guide for Patients and Primary Care Clinicians. Clin. Diabetes.

[42] Gasoyan H., Sarwer D.B. (2022). Addressing insurance-related barriers to novel antiobesity medications: Lessons to be learned from bariatric surgery. Obesity.

[43] Goldkorn M., Schwartz B., Monterosso J. (2025). Views Among the General Public on New Anti-Obesity Medications and on the Perception of Obesity as a Failure of Willpower. Obes. Sci. Pract..

[44] Post S.M., Stock M.L., Persky S. (2025). Comparing the Impact of GLP-1 Agonists vs. Lifestyle Interventions and Weight Controllability Information on Stigma and Weight-Related Cognitions. Int. J. Behav. Med..

[45] Westbury S., Oyebode O., van Rens T., Barber T.M. (2023). Obesity Stigma: Causes, Consequences, and Potential Solutions. Curr. Obes. Rep..

[46] Li Z., Han Z., Sun R., Xuan X., Huang C. (2025). Long-Term Efficacy Trajectories of GLP-1 Receptor Agonists: A Systematic Review and Network Meta-Analysis. Diabetes Metab. Syndr. Obes..

[47] Rodriguez P.J., Zhang V., Gratzl S., Do D., Goodwin Cartwright B., Baker C., Gluckman T.J., Stucky N., Emanuel E.J. (2025). Discontinuation and Reinitiation of Dual-Labeled GLP-1 Receptor Agonists Among US Adults with Overweight or Obesity. JAMA Netw. Open.

[48] Merhi Z., Karekar M., Mouanness M. (2025). GLP-1 receptor agonist for weight loss and fertility: Social media and online perception versus evidence-based medicine. PLoS ONE.

[49] Lee S., Narula N., Weglarz M., Lee J., Kim D. (2025). Evaluation of social media videos on weight-loss surgery and GLP-1 agonists: A content quality and reliability analysis. Surg. Endosc..

[50] The Center for Self-Determination Theory. Perceived Competence Scales (PCS). https://selfdeterminationtheory.org/perceived-competence-scales/.

[51] Rosenstock I.M., Strecher V.J., Becker M.H. (1988). Social learning theory and the Health Belief Model. Health Educ. Q..

[52] Teixeira P.J., Silva M.N., Mata J., Palmeira A.L., Markland D. (2012). Motivation, self-determination, and long-term weight control. Int. J. Behav. Nutr. Phys. Act..

[53] Palmeira A.L., Teixeira P.J., Branco T.L., Martins S.S., Minderico C.S., Barata J.T., Serpa S.O., Sardinha L.B. (2007). Predicting short-term weight loss using four leading health behavior change theories. Int. J. Behav. Nutr. Phys. Act..

[54] Bandura A. (1997). Self-Efficacy: The Exercise of Control.

[55] Samdal G.B., Eide G.E., Barth T., Williams G., Meland E. (2017). Effective behaviour change techniques for physical activity and healthy eating in overweight and obese adults; systematic review and meta-regression analyses. Int. J. Behav. Nutr. Phys. Act..

[56] Strecher V.J., DeVellis B.M., Becker M.H., Rosenstock I.M. (1986). The role of self-efficacy in achieving health behavior change. Health Educ. Q..

[57] World Health Organization WHO Issues Global Guideline on the Use of GLP-1 Medicines in Treating Obesity. https://www.who.int/news/item/01-12-2025-who-issues-global-guideline-on-the-use-of-glp-1-medicines-in-treating-obesity.

[58] Sarpatwari A., Soto M.J., Ganguli I., Sloan C.E., Goss F., Sinaiko A.D. (2025). Glucagon-Like Peptide-1 Receptor Agonist Order Fills and Out-of-Pocket Costs by Race, Ethnicity, and Indication. JAMA Health Forum.

[59] Hwang J.H., Laiteerapong N., Huang E.S., Mozaffarian D., Fendrick A.M., Kim D.D. (2025). Fiscal Impact of Expanded Medicare Coverage for GLP-1 Receptor Agonists to Treat Obesity. JAMA Health Forum.

[60] McCall K.L., Mastro Dwyer K.A., Casey R.T., Samana T.N., Sulicz E.K., Tso S.Y., Yalanzhi E.R., Piper B.J. (2025). Safety analysis of compounded GLP-1 receptor agonists: A pharmacovigilance study using the FDA adverse event reporting system. Expert. Opin. Drug Saf..

[61] Novo Nordisk Tips for Prior Authorization. https://www.novomedlink.com/content/dam/novonordisk/novomedlink/new/obesity/product-information/resources/library/documents/wegovy-tips-for-prior-authorizations.pdf.

[62] Mehrtash F., Dushay J., Manson J.E. (2025). Integrating Diet and Physical Activity When Prescribing GLP-1s-Lifestyle Factors Remain Crucial. JAMA Intern. Med..

[63] Gonzalez-Rellan M.J., Drucker D.J. (2025). The expanding benefits of GLP-1 medicines. Cell Rep..

[64] Gleason P.P., Urick B.Y., Marshall L.Z., Friedlander N., Qiu Y., Leslie R.S. (2024). Real-world persistence and adherence to glucagon-like peptide-1 receptor agonists among obese commercially insured adults without diabetes. J. Manag. Care Spec. Pharm..

[65] Stefan N., Birkenfeld A.L., Schulze M.B., Ludwig D.S. (2020). Obesity and impaired metabolic health in patients with COVID-19. Nat. Rev. Endocrinol..

[66] Aburto S., Cisterna M., Acuña J., Ruíz C., Viscardi S., Márquez J.L., Villano I., Letelier P., Guzmán N. (2022). Obesity as a Risk Factor for Severe COVID-19 in Hospitalized Patients: Epidemiology and Potential Mechanisms. Healthcare.

[67] Xie Y., Choi T., Al-Aly Z. (2025). Mapping the effectiveness and risks of GLP-1 receptor agonists. Nat. Med..

[68] Teixeira P.J., Carraça E.V., Marques M.M., Rutter H., Oppert J.M., De Bourdeaudhuij I., Lakerveld J., Brug J. (2015). Successful behavior change in obesity interventions in adults: A systematic review of self-regulation mediators. BMC Med..

[69] Wing R.R., Phelan S. (2005). Long-term weight loss maintenance. Am. J. Clin. Nutr..

[70] Albasheer O., Hakami N., Abdelwahab S.I., Alqassim A.Y., Alharbi A., Abdelmola A.O., Altraifi A.A.A., Medani I.E., Hakami A.M.S., Moafa M.H. (2024). Utilisation of the health belief model to study the behavioural intentions relating to obesity management among university students: A cross-sectional study. BMJ Open.

[71] (2018). The U.S. Burden of Disease Collaborators. The State of US Health, 1990–2016: Burden of Diseases, Injuries, and Risk Factors Among US States. JAMA.

[72] Carter R., Moodie M. (2010). The Cost-Effectiveness of Obesity Prevention. Obesity Epidemiology: From Aetiology to Public Health.

[73] Silva M.N., Markland D., Carraça E.V., Vieira P.N., Coutinho S.R., Minderico C.S., Matos M.G., Sardinha L.B., Teixeira P.J. (2011). Exercise autonomous motivation predicts 3-yr weight loss in women. Med. Sci. Sports Exerc..

[74] Ukhanova M., Wozny J.S., Truong C.N., Ghosh L., Krause T.M. (2025). Trends in glucagon-like peptide 1 receptor agonist prescribing patterns. Am. J. Manag. Care.

