# Supplementary material for: Considering Glucagon-like Peptide-1 Receptor Agonists (GLP-1RAs) for Weight Loss: Insights from a Pragmatic Mixed-Methods Study of Patient Beliefs and Barriers
Source: Healthcare (Basel). 2026 Jan 12;14(2):186. doi: 10.3390/healthcare14020186 (PMC12841317; doi:10.3390/healthcare14020186)
Supplement: Supplementary file 1 [file healthcare-14-00186-s001.zip › healthcare-4017551-supplementary.pdf]

Group A

S1. Participants considering GLP-1RA interview questions

1. General Awareness and Perception of GLP-1 Medications

- What's your perception of these medications? (Probe: thinking about safety or effectiveness)
    - What have you heard from friends or family about the medication?
    - What is your expectations on wt loss speed?
    - Do you think you'll need to do anything else besides taking the medication to lose weight?

2. Motivation and Reasons for Considering Use

- What would motivate you to start using a GLP-1 medication?
- Many people mention 'food noise' as an issue hindering weight loss. Do you have this…talk about it..
- If you were prescribed a GLP-1 medication, what benefits would you hope to see?
- Are you seeking to make a significant lifestyle change, and would GLP-1 be part of that plan?
- Would this be the first step you have ever taken to try to achieve weight loss? If no, have you ever taken medication?

3. Concerns or Barriers to Use

- What concerns do you have about using GLP-1 medications? Any as a parent or a partner?
- Are you hesitant about injectable medications, and if so, why?
- Do you have any concerns about the long-term safety or side effects of GLP-1 medications?
- What would make you hesitant or reluctant to try GLP-1 medications?

4. Experience with Other Treatments

- What other treatments or lifestyle changes have you tried to manage your health (e.g., diet, exercise, other medications)?

5. Cost and Accessibility

- How important is cost when deciding whether to use a medication like GLP-1?
- How do you feel about the potential need for ongoing prescriptions and doctor visits?

6. Motivation and Reasons for Considering Use

- If you were prescribed a GLP-1, who led discussing this treatment option you or your health care provider?
- Invasiveness ranges from surgery as the most invasive to medication to lifestyle changes for weight loss. Where are you the most comfortable on this spectrum?
- (How important is it for you to have non-invasive treatment option, i.e. surgery?)

7. Personal Health Goals and Values

- How do you define success when it comes to managing your health and wellness?
- What role does medication play in your broader health goals (e.g., managing chronic conditions, preventing disease, improving quality of life)?
- If you were to start a GLP-1 medication, how would you measure its success for you personally?

**8. Long-term Considerations**<br>• Do you think about the long-term commitment involved with medications like GLP-1? How does that affect your decision?<br>• How likely are you to continue using a medication like GLP-1 if it required ongoing use to maintain results?<br>• What would make you discontinue the use of GLP-1 medications if you started taking them?<br>• What did your provider say about long term use?<br>• You mentioned a significant weight loss goal. As you loss weight, this will be noticeable. I'm sure some will comment on your changing size. Do you think you will share with others that you are taking a wt loss medication or not?<br>o If not, can you talk more about that?
Anything else you would like to share with us.
